# 

**Published:** 1910-11-19

**Authors:** 


					Hf IN
?Rv J&\ JmLs Jmn* S>? f?'? 5 f~r?r'f.K*-*>i.rnt- f ftv-*?>
M> ^ * ?^un^ ? ^jFFfO^
HOSPITAL
Telegraphic Address:
Ospedale, London.
THE MODERN MEDICAL
AND
INSTITUTIONAL JOURNAL.
Telephone Number
2734 Gerrard.
HEW SERIES. No. 197, Vol. VIII. GATTTTIT>AV
No. 1269, Vol. XLIX. SATURDAY,
Nov. 19th, 1910.
PRICE THREEPENCE

				

